# Unilateral biportal endoscopic spine surgery: a meta-analysis unveiling the learning curve and clinical benefits

**DOI:** 10.3389/fsurg.2024.1405519

**Published:** 2024-11-07

**Authors:** Shu-Xin Liu, Rui-Song Chen, Chien-Min Chen, Li-Ru He, Shang-Wun Jhang, Guang-Xun Lin

**Affiliations:** ^1^ Department of Orthopedics, Panjin Central Hospital, Panjin, Liaoning, China; ^2^ Department of Orthopedics and Traumatology of Traditional Chinese Medicine, The Third Hospital of Xiamen, Xiamen, China; ^3^ Division of Neurosurgery, Department of Surgery, Changhua Christian Hospital, Changhua, Taiwan; ^4^ Department of Leisure Industry Management, National Chin-Yi University of Technology, Taichung, Taiwan; ^5^ Department of Biomedical Sciences, National Chung Cheng University, Chiayi, Taiwan; ^6^ Department of Anesthesia and Surgery, The First Affiliated Hospital of Xiamen University, Xiamen University, Xiamen, Fujian, China; ^7^ Department of Orthopedics, The First Affiliated Hospital of Xiamen University, School of Medicine, Xiamen University, Xiamen, China; ^8^ The Third Clinical Medical College, Fujian Medical University, Fuzhou, Fujian, China

**Keywords:** unilateral biportal endoscopic, UBE, BESS, lumbar degenerative disease, learning curve

## Abstract

**Objective:**

To provide insights into the learning curve of unilateral biportal endoscopic (UBE) spine surgery by synthesizing available evidence on critical points and associated clinical outcomes.

**Methods:**

A comprehensive literature search was conducted across multiple databases, yielding a pool of relevant studies. Inclusion criteria encompassed studies reporting on UBE learning curves and quantitative data related to clinical outcomes (operative time, hospital stay, and complications).

**Results:**

A total of five studies were included in the analysis, providing six datasets to elucidate the UBE learning curve. Three of the five studies analyzed learning curves using the Cumulative Sum method and identified cutoff points. One study plotted learning curves and determined cutoff points based on surgical time analysis, while the remaining one study (providing two datasets) plotted learning curves using the phased analysis method. The mean value of the cutoff point in terms of the number of cases required to reach proficiency in time to surgery was calculated at 37.5 cases, with a range spanning from 14 to 58 cases. Notably, there was a statistically significant difference in time to surgery between the late group and the early group, with the late group demonstrating a significantly reduced time to surgery (*P* < 0.0001). Additionally, the determined cutoff points exhibited significant variations when applied to patient outcome parameters, including postoperative hospitalization, postoperative drainage, and surgical complications (*P* < 0.05).

**Conclusion:**

While the analysis indicates that UBE surgery's learning curve is associated with surgical time, the limited focus on this metric and potential discrepancies in cutoff point determination highlight the need for a more comprehensive understanding.

## 1 Introduction

In contemporary society, shifts in lifestyle and occupational factors have contributed to a steady rise in the incidence of spinal diseases (1–3). Traditional open surgery has historically served as the primary clinical intervention for the treatment of such spinal conditions (4, 5). While it offers distinct advantages such as comprehensive decompression and extensive visualization of the surgical site, it is not without notable shortcomings. These include the inherent drawbacks of substantial surgical trauma, protracted operative durations, and an elevated risk profile for postoperative complications (6–8). In contrast to traditional open surgery, minimally invasive surgical techniques offer the potential to mitigate the aforementioned drawbacks associated with open surgical procedures (9–11). Consequently, the realm of minimally invasive spine surgery has garnered increasing attention and interest among a growing cohort of scholars and medical practitioners (12–14).

The unilateral biportal endoscopic (UBE) techniques, representing a domain of minimally invasive spinal surgery, have experienced noteworthy advancements in recent years (15–19). In concert with the evolution of minimally invasive surgical paradigms and the continuous refinement of spinal endoscopic instrumentation, the UBE technique has garnered escalating attention within the clinical domain (16, 17). The UBE technique represents a convergence of principles derived from microendoscopy and single-channel spinal endoscopy, thus amalgamating the respective strengths of these approaches to engender a distinctive set of advantages (20, 21). The UBE technique is characterized by the utilization of two primary channels during its execution. The first of these channels serves as the observation conduit and is typically equipped with a 0° or 30° arthroscope (15, 22). The second channel is designated as the operating channel and, can hold specialized instruments customized specifically for the UBE or open surgical instruments (23, 24). It is noteworthy that these specialized instruments can also be employed in conjunction with conventional spinal surgical instrumentation, enhancing the versatility and applicability of the UBE approach (25, 26). Consequently, the clinical application of the UBE technique has proliferated significantly. This upsurge in utilization underscores the growing recognition and adoption of UBE as a valuable approach within the clinical domain. Nevertheless, notwithstanding the evident advantages associated with the minimally invasive attributes of the UBE approach, it is noteworthy that a substantial proportion of spine surgeons remain unacquainted with this technique. Moreover, mastering the UBE method entails a protracted and intricate learning curve (27). Consequently, some surgeons, even those possessing considerable expertise in the field, may exhibit reluctance to engage in the learning process, occasionally dismissing UBE as minimally effective or a mere semblance of a surgical procedure.

Therefore, it is crucial for the medical community to understand the UBE learning curve and implement effective training programs to facilitate clinical proficiency. However, there is still no consensus on the characteristics of this learning curve or the strategies needed to improve it. The primary aim of this study is to systematically review the learning curve of UBE surgery, quantify the number of cases required to achieve technical proficiency, and discuss strategies to optimize the learning process.

## 2 Methods

### 2.1 Search strategy

To procure pertinent literature, an exhaustive search was undertaken by the authors across multiple databases, including PubMed, Embase, Web of Science, Cochrane Library, and the China National Knowledge Infrastructure database. To ensure comprehensive coverage, a set of meticulously chosen search terms were employed. These search terms encompassed “learning curve,” “training curve,” “endoscopy,” “unilateral biportal,” “biportal endoscopy,” “UBE,” and “BESS.” Both textual and Medical Subject Headings (MeSH) terms were thoughtfully amalgamated in order to optimize sensitivity and inclusivity, with the focus on identifying human studies across languages. Additionally, to augment the scope of the search, a manual examination of reference lists was conducted to identify any further primary studies of relevance.

### 2.2 Data extraction

The screening criteria applied in this study comprised the following inclusion prerequisites: (1) Inclusion of studies in both the English and Chinese languages; (2) Encompassing investigations concerning human patients that reported on the learning curves associated with UBE techniques; (3) Incorporation of all categories of observational studies, including randomized and non-randomized controlled trials; and (4) Availability of quantitative data pertaining to clinical outcome parameters.

Data extraction was undertaken by two independent reviewers, each responsible for extracting data from rigorously screened reports. Any disparities encountered during this process were meticulously addressed through a consensus-driven resolution following a comprehensive discussion.

Learning curve studies typically assess the number of cases or time required for a surgeon to achieve proficiency in a new technique and evaluate improvements in technical performance. Common methods used include: (1) Cumulative Sum (CUSUM) Analysis: A statistical approach that tracks performance changes by accumulating deviations between each surgical outcome and a predefined standard, thus plotting the learning curve; (2) Surgical Time Analysis: This method evaluates the learning curve by analyzing how surgical duration changes with increased procedure volume; (3) Phased Analysis: The learning process is divided into distinct phases (e.g., early and late phases), allowing comparisons of performance metrics across different stages of experience.

The data extracted included key parameters such as the study design, statistical methods, sample size, patient demographics (e.g., age, gender, operative levels, and diagnosis), surgical duration, length of hospitalization, and complication rates. In this study, the threshold marking the onset of the learning curve was explicitly defined and identified.

### 2.3 Quality evaluation

For the purpose of quality assessment, each study incorporated in this investigation underwent a comprehensive evaluation employing the Newcastle-Ottawa Scale (NOS) (28). This scale consists of eight items divided into three domains: selection, comparability, and outcomes for cohort studies, or exposure for case-control studies. The comparability domain assesses the alignment between cases and controls based on study design and analysis. The exposure domain evaluates the determination of exposure or outcomes, accounting for nonresponse rates where applicable (29).

Studies that received five stars or more on the NOS were included in this review, ensuring a high standard of quality to enhance the reliability of the meta-analysis outcomes (30).

### 2.4 Statistical analysis

All statistical analyses were conducted utilizing Statistical Analysis Review Manager version 5.3.5, a software resource developed by the Cochrane Collaboration, Oxford, United Kingdom. The patient cohort was stratified into distinct early and late groups, a demarcation predicated upon the investigator's stipulated cut-off number. Specifically, the early group encompassed patients treated prior to the delineated cutoff point, while the late group comprised patients undergoing treatment subsequent to this threshold. While certain investigators employed multiple group divisions, it became evident that the most salient disparities manifested between the initial patient cohort and the subsequent groups.

The presentation of results adheres to the utilization of forest plots, thereby encapsulating statistical estimates, 95% confidence intervals (CI), and relative weights denoted as the proportional size of the central square, the horizontal line, and the square. Dichotomous variables in the comparative research were subjected to assessment using odds ratios (OR) or risk ratios, while continuous variables were evaluated using weighted mean differences (WMD) or standard mean differences. To gauge the presence of heterogeneity among the studies, we conducted both the chi-squared (*χ*2) and *I*^2^ tests. In instances where the *p*-value exceeded 0.1 or *I*^2^ was below 50%, we regarded the studies as homogeneous and subsequently employed a fixed-effects model. Conversely, when *I*^2^ surpassed the threshold of 50%, we opted for a random-effects model. Statistical significance was established at a *p*-value of less than 0.05.

Publication bias was evaluated using visual inspection of funnel plots and statistically using Egger's test.

## 3 Results

### 3.1 Study characteristics

**Figure 1:**
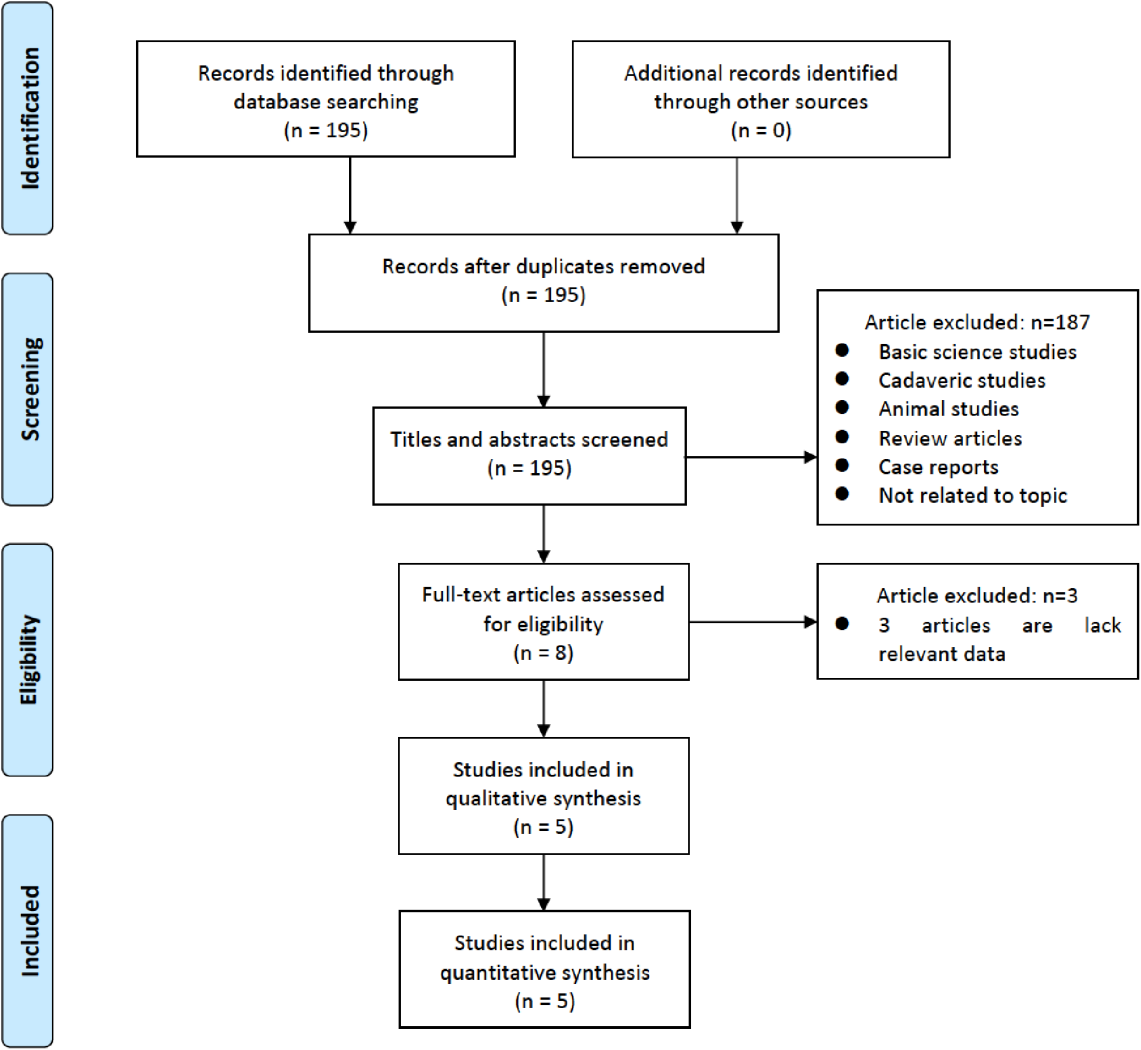
Flowchart of study selection for meta-analysis.

The flowchart detailing the inclusion process of the studies is illustrated in Figure 1. Initially, 195 reports were identified through an extensive literature search. After screening titles and abstracts, 190 studies were excluded. Upon further review of the full-text articles, five studies met the predefined inclusion criteria and were included in the final analysis (26, 31–34). One of these studies presented two distinct datasets (35).

All included studies employed a cohort-based design. Patients were categorized into early and late groups based on the cumulative surgical experience of the surgeons performing UBE. Notably, all five studies were retrospective in nature, with each surgery conducted by a single surgeon. Geographically, three studies originated from China, and two from Korea. The cohort consisted of 542 patients, including 257 females and 285 males. The L4–5 spinal segment was the most frequently treated level, and the mean age of the patients was 60.6 years (Table 1).

**Table 1:** Characteristics of the included studies.

Study (year)	Study design	Country	Number(M/F)	Mean age (range), years	BMI (range), kg/m^2^	Operation level	Diagnosis
Chen 2022 (26)	Retrospective	China	97 (52/45)	51.5 ± 15.4 (21-86)	23.9 ± 4.8 (16.1–31.6)	L3–4 (9); L4–5 (40); L5–S1 (48)	LDH (97)
Chio 2016 (31)	Retrospective	Korea	68 (28/40)	58.0 ± 15.3 (23–85)	NR	NR	LDH (25), revision LDH (3);stenosis (39); Synovial cyst (1)
Park 2019 (32)	Retrospective	Korea	60 (31/29)	67.6 (41–91)	24.8 (16.3–33.1)	L2–3 (5); L3–4 (5); L4–5 (44); L5–S1 (6)	Stenosis (60)
Wang-1 2022 (33)	Retrospective	China	60 (36/24)	56.4 ± 16.0	NR	L2–3 (1); L3–4 (7); L4–5 (33); L5–S1 (19)	LDH (60)
Wang-2 2022 (33)	Retrospective	China	60 (31/29)	65.3 ± 13.0	NR	L1–2 (2); L2–3 (3); L3–4 (11); L4–5 (38); L5–S1 (6)	Stenosis (60)
Xu 2022 (34)	Retrospective	China	197 (107/90)	64.83 ± 14.29 (34–91)	21.89 ± 2.23	L3–4 (16); L4–5 (115); L5–S1 (66)	LDH (90); stenosis (107)

BMI, body mass index; LDH, lumbar disc herniation; NR, not report.

Three of the five studies analyzed the learning curve using the CUSUM method, identifying specific cutoff points (Table 2). One study used surgical time analysis to determine the cutoff point, while the remaining one (providing two datasets) utilized phased analysis (Table 2). The study found that the learning curve for surgeons varied, with a mean of 37.5 cases (range: 14–58) needed to reach a plateau of surgical proficiency.

**Table 2:** Summary of outcome data.

Study (year)	Cases (*n*)	Cutoff point (*n*)	Observation method
Chen 2022 (26)	97	24	CUSUM
Chio 2016 (31)	68	14	Comparative (surgical time analysis)
Park 2019 (32)	60	58	CUSUM
Wang-1 2022 (33)	60	NR	Comparative (phased analysis)
Wang-2 2022 (33)	60	NR	Comparative (phased analysis)
Xu 2022 (34)	197	54	CUSUM

CUSUM, cumulative sum analysis; NR, not report.

### 3.2 Quality analysis and publication bias

The comprehensive results of the risk of bias assessment are presented in Table 3. It is noteworthy that all the studies under review received a rating of five stars or more in their respective assessments, indicative of the overall good quality of the reviewed studies.

**Table 3:** Quality assessment of the included studies.

Studies	Selection				Comparability	Exposure			Total scores (of 9)
	Is the case definition adequate?	Representativeness of the cases	Selection of controls	Definition of controls	Comparability of cases and controls on the basis of the design or analysis	Ascertainment of exposure	Same method of ascertainment for cases and controls	Non-response rate	
Chen 2022 (26)	☆	☆			☆☆	☆	☆	☆	7
Chio 2016 (31)	☆	☆			☆☆	☆	☆		6
Park 2019 (32)	☆	☆			☆☆	☆	☆	☆	7
Wang-1 2022 (33)	☆	☆			☆☆	☆	☆		6
Wang-2 2022 (33)	☆	☆			☆☆	☆	☆		6
Xu 2022 (34)	☆	☆			☆☆	☆	☆	☆	8

The “stars” represent the quality rating of the studies based on specific criteria. Each star corresponds to a point that indicates the level of quality in three main domains: selection, comparability, and exposure. A study can earn a maximum of nine stars, with higher ratings indicating better methodological quality.

**Figure 2:**
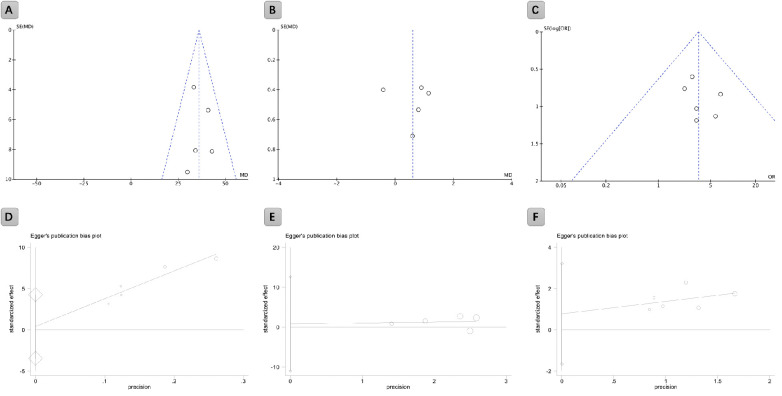
Funnel plot of publication bias for operative time **(A)**, length of hospitalization **(B)**, and complications **(C)** Egger's test for publication bias: operative time **(D)**, length of hospitalization **(E)**, and complications **(F)** Egger's test yielded non-significant results for all groups (*P* > 0.05).

Publication bias was assessed using funnel plots for operative time, length of hospitalization, and complications (Figures 2A–C). The scatter patterns in the funnel plots were generally symmetrical, suggesting a low risk of publication bias. Additionally, Egger's test results showed *p*-values greater than 0.05 for all outcomes (Figures 2D–F), further supporting the absence of significant publication bias.

### 3.3 Operative time (mins)

**Figure 3:**
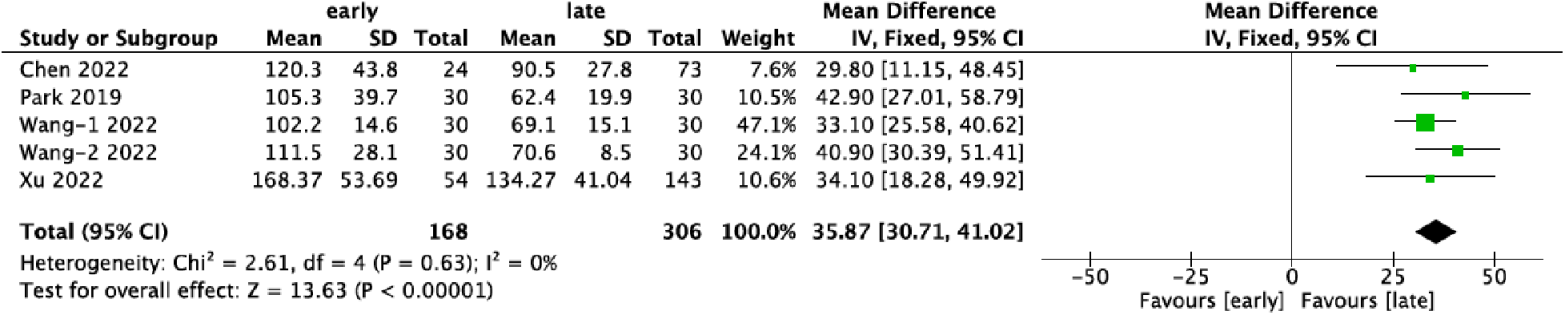
Forest plots comparing operative time in the early and late groups.

Quantitative data on operative time were provided by five datasets, encompassing a total of 474 patients. Of these, 35.4% (168/474) were assigned to the early-experience group, while 64.6% (306/474) were categorized in the late-experience group. The mean duration of surgery in the late-experience group (85.4 min) was significantly shorter than in the early-experience group (121.5 min), with a difference of 36.1 min (WMD: 35.87; 95% CI: 30.71–41.02; *I*² = 0%; *P* < 0.0001; Figure 3). The forest plot demonstrates a consistent reduction in operative time as experience increases, with low variability and no outliers, reinforcing the conclusion that technical proficiency improves surgical efficiency.

### 3.4 Hospital stay (days)

**Figure 4:**
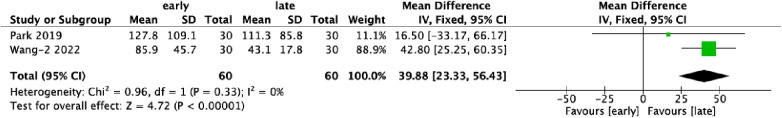
Forest plots comparing length of hospital stays in the early and late groups.

Five datasets provided data on the duration of hospitalization, encompassing 474 patients, with 35.4% (168/474) in the early-experience group and 64.6% (306/474) in the late-experience group. The mean hospital stay in the late-experience group was significantly shorter compared to the early-experience group (WMD: 0.59; 95% CI: −0.01–1.20; *I*² = 53%; *P* = 0.05; Figure 4). The forest plot indicates a trend towards shorter hospital stays in the late-experience group, though moderate variability was observed across studies.

### 3.5 Postoperative drainage (ml)

**Figure 5:**
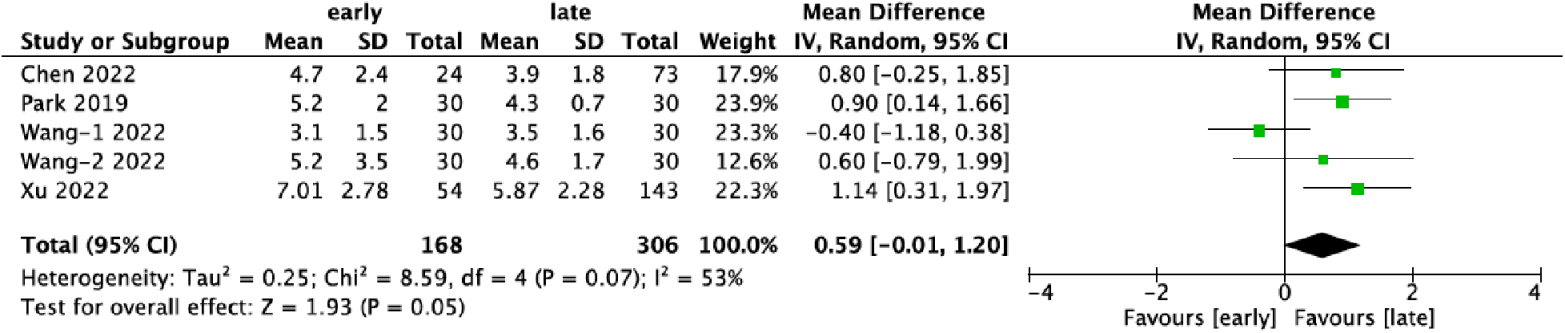
Forest plots comparing postoperative drainage in the early and late groups.

Two studies reported data on postoperative drainage, involving 120 patients, evenly distributed between the early- and late-experience groups. Analysis revealed a significant reduction in postoperative drainage in the late-experience group compared to the early-experience group (WMD: 39.88; 95% CI: 23.33–56.43; *I*² = 0%; *P* < 0.0001; Figure 5). The forest plot shows a strong, consistent trend toward reduced postoperative drainage with no variability or outliers, suggesting that greater experience leads to better hemostasis and tissue management, improving postoperative recovery.

### 3.6 Complications

**Figure 6:**
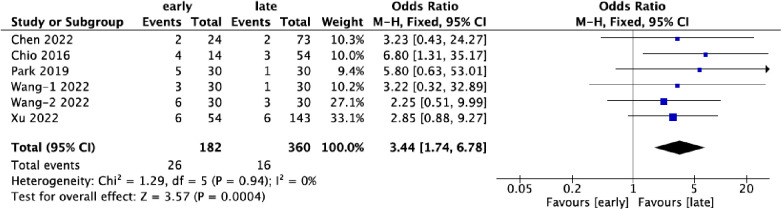
Forest plots comparing complications in the early and late groups.

Six datasets contributed data on complications associated with UBE surgery, covering 542 patients. Of these, 33.6% (182/542) belonged to the early-experience group, while 66.4% (360/542) were in the late-experience group. The rate of complications was significantly higher in the early-experience group (14.3%, 26/182) compared to the late-experience group (4.4%, 16/360) (OR: 3.44; 95% CI: 1.74–6.78; *I*² = 0%; *P* = 0.0004; Figure 6). The forest plot highlights a clear trend towards fewer complications in the late-experience group, with no heterogeneity or outliers. The odds of complications in the early-experience group were more than three times higher, underscoring the importance of experience in reducing surgical risks and improving patient outcomes.

## 4 Discussion

In recent years, the application of UBE has gained increasing prominence in the treatment of lumbar degenerative diseases and other spinal disorders. This surgical modality has garnered recognition for its merits, encompassing a broad field of view, surgical flexibility, reduced tissue trauma, effective nerve decompression, and expedited postoperative recovery (36, 37). However, even proficient spine surgeons encounter substantial challenges and inherent risks during the initial phases of adopting UBE surgery into their practice. The introduction of a new surgical technique into clinical practice necessitates a meticulous examination of its learning curve. Such a study serves the paramount purpose of expediting the surgeon's proficiency in adopting the technique and facilitating judicious surgical procedure selection (38). However, it is imperative to underscore the conspicuous absence of a comprehensive meta-analysis addressing the learning curve associated with UBE surgery for the treatment of lumbar degenerative diseases. This notable gap in the existing body of research underscores the urgent requirement for comprehensive meta-analytical investigations in this domain.

### 4.1 Outcome measures and cutoff point

The learning curve typically encompasses three fundamental components: the initial point marking the inception of the first surgical case, the learning rate characterizing the rate of skill acquisition, and the asymptote representing the attainment of an expert level, denoting the juncture where the learning curve levels off (39). This phenomenon implies that the learning process is intricate and demanding. A paramount objective of learning curve studies is to delineate a tipping point or transitional phase that distinguishes between the early training phase and the subsequent, more seasoned phase in a surgeon's experience (40). As a critical mass of cases is accrued, discernible reductions in operative time and operative complications emerge, accompanied by notable correlations with improved clinical outcomes.

Previous studies often used a single variable, such as surgical time, to analyze the learning curve. While convenient, this method is somewhat subjective and lacks the precision needed to determine the exact number of cases required for proficiency. In contrast, the CUSUM method, when combined with curve fitting techniques, provides a more objective depiction of the learning process. In this meta-analysis, three studies (three datasets) employed the CUSUM method, while two (three datasets) relied on dichotomous comparisons between early and late experience groups. The average threshold for differentiating between these groups was 37.5 cases, with a range of 14–58 cases across the studies.

### 4.2 Strategies to expedite the learning curve

Several strategies can expedite the learning curve for UBE surgery, based on empirical evidence and clinical insights: (1) Comprehensive training program: A structured and comprehensive training program is crucial for rapid skill acquisition in UBE (41, 42). Such programs should include theoretical and conceptual modules covering the principles of UBE, alongside hands-on workshops, cadaveric dissection, and observation of live surgeries. Microsurgical training plays an especially important role in refining the tactile and bimanual dexterity required for navigating complex neural and vascular structures. Given the intricate nature of UBE, a well-organized training curriculum is essential for achieving proficiency. Moreover, early mentorship from a physician experienced in UBE can help reduce complications; (2) Rigorous patient selection: Prudent patient selection is pivotal in ensuring clinical success and simultaneously expediting the learning process (43). Surgeons embarking on their UBE journey are advised to adhere rigorously to established indications for the procedure, especially during the early stages of skill development. UBE should ideally be reserved for typical and uncomplicated cases until the learning curve reaches a plateau. Once a sufficient level of competence is achieved, surgeons can gradually extend their repertoire to encompass more intricate and challenging cases. The meticulous selection of patients aligns the procedure with clinical appropriateness and streamlines the learning curve by limiting the complexities encountered during the initial phases.

### 4.3 Complications and preventions of UBE

Although UBE offers numerous benefits as a minimally invasive spinal surgery technique, it is not without potential complications. A thorough understanding of these complications and strategies for their prevention is essential for optimizing patient outcomes. Common complications include epidural hematomas, nerve root or dural injuries, incomplete decompression, recurrence, and soft tissue damage (15, 44). In a comprehensive analysis involving 797 patients who underwent UBE procedures, postoperative complications were identified in 10.3% of cases, with epidural hematoma and incomplete decompression emerging as the most prevalent issues (45). UBE's unique requirement for two surgical channels and continuous saline irrigation can result in varying degrees of soft tissue damage, particularly when operative times are extended, or irrigation pressures are elevated. To minimize these risks, it is essential to regulate saline flow rates and pressure while maintaining clear visualization during the surgery (46). Additionally, dural and nerve injuries are often caused by the use of drills and forceps. Immediate intervention after such injuries can prevent severe neurological sequelae (47, 48).

Thorough preoperative planning, comprehensive knowledge of spinal anatomy, and careful intraoperative observation are essential to minimize the risk of nerve injury (46). Utilizing advanced imaging modalities, such as intraoperative navigation systems, can improve the accuracy of UBE procedures. A thorough preoperative assessment of vascular anatomy, including abnormalities or variations, can guide the safe placement of trocars and minimize the risk of vascular injury (49). Surgeons should take extra care when working in the vicinity of vascular structures and use meticulous hemostatic techniques when necessary. Gentle handling of neural structures, precise instrumentation control, and a thorough understanding of spinal anatomy are key to preventing dural tears. Adequate cerebrospinal fluid drainage, when necessary, can help reduce cerebrospinal fluid pressure and mitigate the risk of dural injury (34). Strict adherence to aseptic surgical technique, including thorough preoperative skin preparation and toweling, as well as perioperative antibiotic prophylaxis, can greatly reduce the risk of surgical site infection (50, 51). Ensuring a sterile surgical environment and proper wound closure is also critical (52). Effective hemostasis and meticulous closure of tissue layers during surgery can minimize the risk of postoperative hematoma or seroma (31). If necessary, appropriate wound drainage should be considered to prevent fluid accumulation (53). Routine maintenance and calibration of endoscopic instruments is imperative to prevent technical malfunction during the procedure. The surgeon should also take care to avoid inadvertent damage to the instruments to ensure their proper function (50). Effective postoperative pain management strategies should be implemented, including tailored pain management plans and patient education about what to expect after surgery (51, 54). Appropriate positioning and support during surgery can also reduce postoperative discomfort. Regular maintenance of instruments, careful intraoperative handling, and selection of appropriate instruments for specific tasks can help prevent instrument breakage or failure.

### 4.4 Study limitations

Several limitations should be considered when interpreting the findings of this study: First, the majority of included studies were retrospective, which may introduce significant heterogeneity. This variability complicates the interpretation of results, as differences in study design and methodology may have affected the outcomes. Second, surgical outcomes can vary substantially between surgeons at different skill levels. Unfortunately, this study could not assess the baseline proficiency of the surgeons involved, adding complexity to the analysis. Third, the learning curve for UBE may vary depending on whether it is used for treating disc herniation or spinal stenosis, as the technical demands of these procedures differ. Lastly, the determination of cutoff points lacked a standardized basis, contributing to some uncertainty in the findings.

## 5 Conclusions

This meta-analysis of the learning curve for UBE surgery identified an average critical threshold of 37.5 cases (range: 14–58 cases) required to reach technical proficiency. Significant differences were observed between early and late-stage surgeons, particularly in operative time, hospitalization, and complications. Operative time reflects procedural efficiency, but it does not account for critical factors such as patient outcomes, recovery, pain management, and long-term quality of life. In some instances, the critical point derived from operative time may not represent the true plateau of the learning curve, where surgeons achieve maximum proficiency.

Future studies should incorporate patient-centered outcome measures, such as long-term recovery, complication rates, and patient satisfaction, in order to provide a more comprehensive understanding of the learning curve. This approach will not only enhance surgical training but also improve patient outcomes in UBE and other endoscopic spine surgeries.

## Data Availability

The original contributions presented in the study are included in the article/Supplementary Material, further inquiries can be directed to the corresponding authors.
